# In Vitro Antioxidant Activities of Enzymatic Hydrolysate from *Schizochytrium* sp. and Its Hepatoprotective Effects on Acute Alcohol-Induced Liver Injury In Vivo

**DOI:** 10.3390/md15040115

**Published:** 2017-04-10

**Authors:** Xixi Cai, Ana Yan, Nanyan Fu, Shaoyun Wang

**Affiliations:** 1 The Key Lab of Analysis and Detection Technology for Food Safety of the MOE, College of Chemistry, Fuzhou University, Fuzhou 350108, China; caixx_0123@163.com (X.C.); nanyan_fu@fzu.edu.cn (N.F.); 2 College of Biological Science and Technology, Fuzhou University, Fuzhou 350108, China; m18144065085@163.com

**Keywords:** *Schizochytrium*, protein hydrolysate, antioxidant, hepatoprotective effects, alcohol-induced liver injury

## Abstract

*Schizochytrium* protein hydrolysate (SPH) was prepared through stepwise enzymatic hydrolysis by alcalase and flavourzyme sequentially. The proportion of hydrophobic amino acids of SPH was 34.71%. The molecular weight (MW) of SPH was principally concentrated at 180–3000 Da (52.29%). SPH was divided into two fractions by ultrafiltration: SPH-I (MW < 3 kDa) and SPH-II (MW > 3 kDa). Besides showing lipid peroxidation inhibitory activity in vitro, SPH-I exhibited high DPPH and ABTS radicals scavenging activities with IC_50_ of 350 μg/mL and 17.5 μg/mL, respectively. In addition, the antioxidant activity of SPH-I was estimated in vivo using the model of acute alcohol-induced liver injury in mice. For the hepatoprotective effects, oral administration of SPH-I at different concentrations (100, 300 mg/kg BW) to the mice subjected to alcohol significantly decreased serum alanine aminotransferase (ALT) and aspartate aminotransferase (AST) activities and hepatic malondialdehyde (MDA) level compared to the untreated mice. Besides, SPH-I could effectively restore the hepatic superoxide dismutase (SOD), catalase (CAT), and glutathione peroxidase (GSH-Px) activities and glutathione (GSH) level. Results suggested that SPH was rich in biopeptides that could be exploited as antioxidant molecules against oxidative stress in human body.

## 1. Introduction

*Schizochytrium* sp., a kind of heterotrophic marine fungus, is well known for the production of Ω-3 fatty acids, pigments, proteins, polysaccharides, etc. [1,2]. A number of researchers have focused on the industrial production of docosahexaenoic acid for *Schizochytrium* sp. studies [3]. However, there is little information on the utilization of *Schizochytrium* sp. byproduct. In addition to a high content of fat, *Schizochytrium* sp. also contains a high amount of protein, which is about 40% (dry weight). Therefore, great efforts are needed to transform these biological wastes into value-added bioproducts. Thus, the utilization of protein recovered from the defatted byproduct presents an opportunity to develop pharmaceutical products and food ingredients. 

Free radicals such as the superoxide anion radical (O_2_**·**^−^) and hydroxyl radical (**·**OH) are highly reactive oxygen species (ROS) with single and unpaired electrons that are involved in biological oxidation process and can cause many adverse effects on food and biological systems [4]. In human organs, free radicals, which are inevitably produced through oxidative metabolism, can induce several diseases such as arteriosclerosis and cancer. Liver injury is a widespread disease that can be caused by an overload of xenobiotics, such as alcohol, CCl_4_, and bromobenzene. Alcohol-induced liver injury has been one of the most frequent causes of liver diseases. The mechanism of liver dysfunction induced by alcohol is thought to involve the generation of free radicals, oxidative stress, and lipid peroxidation [5,6]. More attention has been paid to search for safe antioxidants for effective therapy of oxidative stress-induced diseases. Small molecules with strong antioxidant activities from plants [7,8] and algal [9] have been widely investigated. In addition, preparation of bioactive peptides from proteins through enzymatic hydrolysis has been a hot topic. Peptides from the hydrolysates of Alaska Pollock skin collagen [10], egg white protein [11], chickpea protein [12], and algae protein waste [13] have been prepared and shown to possess antioxidant activities in different oxidation systems.

In this study, *Schizochytrium* sp. byproduct protein hydrolysate was prepared by stepwise enzymatic hydrolysis. The in vitro antioxidant activities of the enzymatic hydrolysates and the hepatoprotective effects on acute alcohol-induced liver injury in vivo were evaluated. The present study suggests that *Schizochytrium* protein hydrolysates have the potential in increasing resistivity against oxidative stress in the human body.

## 2. Results and Discussion

### 2.1. Analyses of Amino Acid Composition and Molecular Weight Distribution of SPH

*Schizochytrium* sp. protein isolates (SP) were enzymatically hydrolyzed by alcalase and flavourzyme sequentially for the preparation of antioxidant peptides. It has been recognized that the amino acid composition of the peptides plays critical roles in their antioxidant activities. The amino acid composition of SPH was determined by amino acid automatic analyzer. Results showed that SPH was rich in Glx and Asx, which accounted for 17.66% and 15.89%, respectively (Table 1). In addition, the total hydrophobic amino acids content in SPH constituted 34.71%. Udenigwe et al. [14] indicated that acidic amino acids such as Glu and Asp contributed to the antioxidant activities of peptides due to the presence of excess electrons which could be donated during interaction with free radicals. For protein hydrolysates and peptides, an increase in hydrophobicity would increase their interaction with lipid targets or entry of the peptides into target organs through hydrophobic associations, which was good for enhancing their antioxidant effects [15,16,17]. In addition, SPH contained 5.79% Lys and 7.81% Arg. Reports have demonstrated that peptides containing amino acids with carboxyl or amino side chains, such as Glu, Gln, Lys, and Arg, could donate electrons or hydrogen atoms to interact with pro-oxidants and inactivate their activity [18,19,20]. Moreover, the amino acids that contained nucleophilic sulphur-containing side chains (Met and Cys), aromatic side chains (Phe and Tyr), or imidazole-containing side chains (His) could donate electron to convert radicals into stable molecules [21]. 

**Figure 1:**
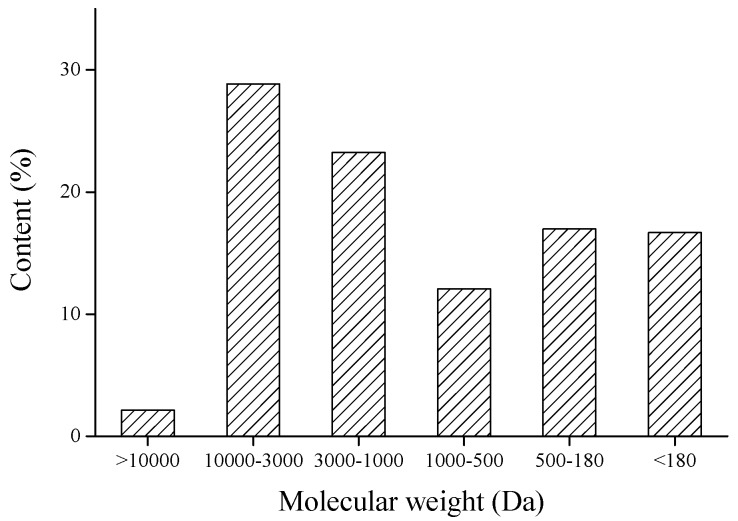
Molecular mass distribution of SPH.

Besides amino acid composition, the molecular weight of peptides is also a significant factor that reflects the antioxidant activities of peptides. MW distribution of SPH was determined using HPLC and the results are shown in Figure 1. The fraction of peptides with MW ranging from 180 to 3000 Da was abundant in SPH, accounting for 52.29%. There are several reports suggesting that peptides with low MW have stronger antioxidant activities than their high MW counterparts. In fact, peptides with low MW could cross the intestinal barrier and further exert their antioxidant effects [22,23].

### 2.2. In Vitro Antioxidant Activities of SPH and Its Fractions

The degrees of hydrolysis (DHs) and DPPH radical scavenging activity of hydrolysate were studied at different hydrolysis stages (Data not shown). The DPPH radical scavenging activity increased from 12.16% (SP) to 38.08% after the first step of hydrolysis by alcalase and the DH reached 8.37%. The activity was further enhanced to 58.06% at the second step of hydrolysis by flavourzyme with a DH of 21.48%. 

In order to study the effect of MW on the antioxidant activities of the peptides, SPH was further fractionated by ultrafiltration to obtain SPH-I (MW < 3 kDa) and SPH-II (MW > 3 kDa). To evaluate the antioxidant activities of SPH and its fractions in vitro, different antioxidant parameters were obtained.

#### 2.2.1. Free Radical Scavenging Activities

**Figure 2:**
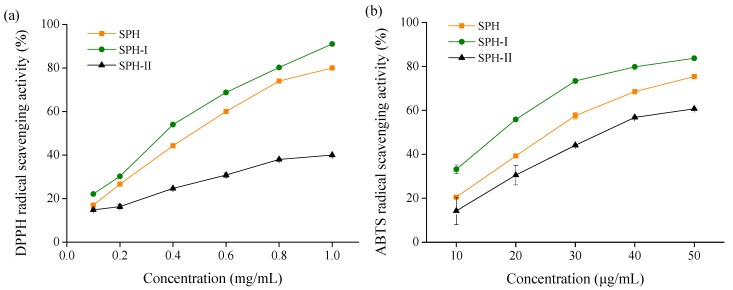
Free radical scavenging activities of SPH and its fractions. (**a**) 1,1-diphenyl-2-picrylhydrazyl (DPPH) radical scavenging activity; (**b**) 2,2′-azinobis-3-ethylbenzthiazoline-6-sulphonate (ABTS) radical scavenging activity.

The ability of SPH and its fractions to scavenge DPPH and ABTS radicals is shown in Figure 2. DPPH and ABTS radicals scavenging activities of SPH and its fractions increased in a concentration dependent manner. SPH-I (MW < 3 kDa) had higher DPPH and ABTS radicals scavenging activities than SPH and SPH-II (MW > 3 kDa) at the same concentration. A lower IC_50_ value was indicative of higher scavenging activity, and the IC_50_ values of SPH-I against DPPH and ABTS radicals were 350 μg/mL and 17.5 μg/mL, respectively. These results indicated that free radical scavenging activities of peptides were related to their MW. Similar results were reported by Li et al. [12], who found that the fraction with low MW of chickpea protein hydrolysate had the highest DPPH radical scavenging activity compared to other fractions. In addition, the peptides with MW < 1 kDa from egg white protein hydrolysate and ethanol-soluble proteins hydrolysate of the *Sphyrna lewini* muscle were found to exhibit higher antioxidant activities than the high MW fractions [11,24].

#### 2.2.2. Reducing Power

**Figure 3:**
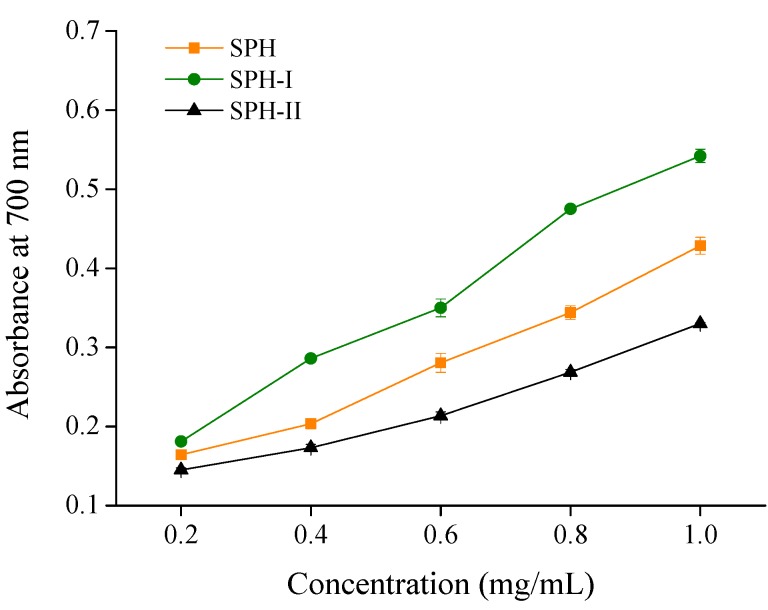
Reducing power of SPH and its fractions.

Reducing power was measured to evaluate the capacity of compounds to donate electrons or hydrogen atoms, and was related to their ability to inhibit the transformation of Fe^3+^ to Fe^2+^ [21,23]. The reducing power of SPH and its fractions was determined and the results are shown in Figure 3. SPH-I had the highest reducing power as compared with SPH and SPH-II in a concentration dependent manner. At the concentration of 1 mg/mL, the absorbance at 700 nm of SPH, SPH-I, and SPH-II was 0.43, 0.54, and 0.33, respectively. This result suggested that all three fractions have the potential to react with free radicals and block radical chain reactions. 

#### 2.2.3. Inhibition of Linoleic Acid Peroxidation

**Figure 4:**
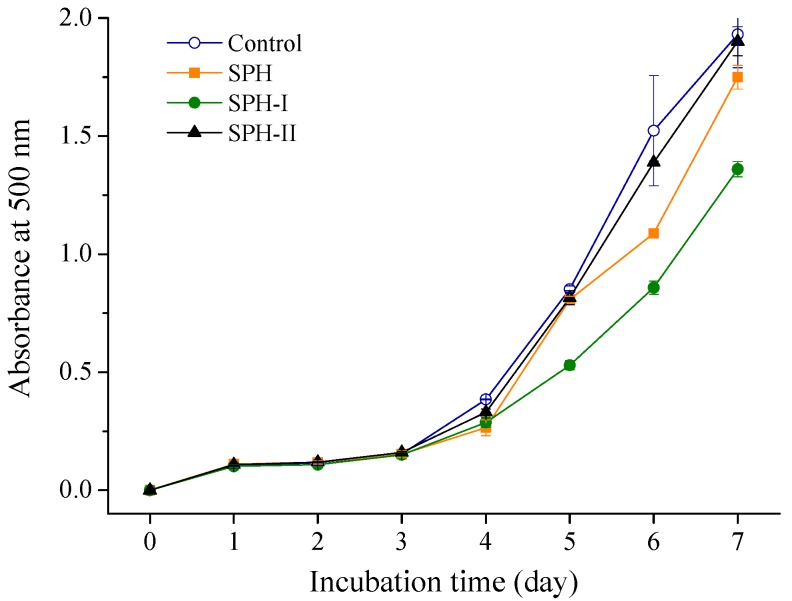
Inhibition activity of SPH and its fractions on linoleic acid peroxidation.

Lipid peroxidation was thought to proceed via radical mediated abstraction of hydrogen atoms from methylene carbons in polyunsaturated fatty acids [16]. The process of lipid peroxidation generated a series of potentially toxic substances such as electrophilic aldehydes and ketones [25,26]. The inhibitory ability of SPH and its fractions on lipid peroxidation was determined in a linoleic acid system. As shown in Figure 4, the control had the highest absorbance at 500 nm, indicating the highest oxidation degree, while the samples with SPH and its fractions (1 mg/mL) could lower the absorbance. SPH-I exhibited the strongest lipid peroxidation inhibition activity, which was in accordance with the previous report [27] showing that low MW peptides were more effective against linoleic acid peroxidation. The lipid peroxidation inhibition activity of SPH and the ultrafiltration fractions may be related to the high content of hydrophobic amino acids, molecular size, and the amino acid residues at the terminal end of the peptides [28].

### 2.3. Effects of SPH-I on Acute Alcohol-Induced Liver Injury in Mice

Various pathways involving multiple types of enzymes and oxidative stress were thought to be associated with the pathological process of alcohol-induced liver injury [6,29]. Oxidative stress, caused by partially-reduced ROS such as superoxide anion (O_2_·^−^), hydroxyl free radical (∙OH), and hydrogen peroxide (H_2_O_2_), played a part in the pathogenesis of alcohol-induced liver injury [25,30]. To study the antioxidant effect of SPH-I in vivo, the well-described alcohol-induced mice hepatotoxicity was used. Alcohol administration was likely to enhance production of free radicals that would initiate lipid peroxidation and decreased activities of antioxidative enzymes, leading to cell membrane damage, intracellular enzyme leakage, and even cell necrosis [18,31].

In this study, forty male Kunming (KM) mice were randomly divided into four groups of ten mice each. Group I served as the normal control and group II was the alcohol model group. Group III and IV were mice treated with SPH-I at 100 and 300 mg/kg BW, respectively, for 24 consecutive days. At the end of the experiment, the mice were euthanized and related biochemical indices were measured.

#### 2.3.1. Effects of SPH-I on Serum ALT and AST Activities

ALT is a cytosolic enzyme that mainly exists in the liver, while AST is primarily present in mitochondria and cytoplasm in the liver. Once hepatocytes are damaged, ALT and AST will leak through the liver cell membrane into circulation and the levels of these enzymes will increase in the serum [30]. 

**Figure 5:**
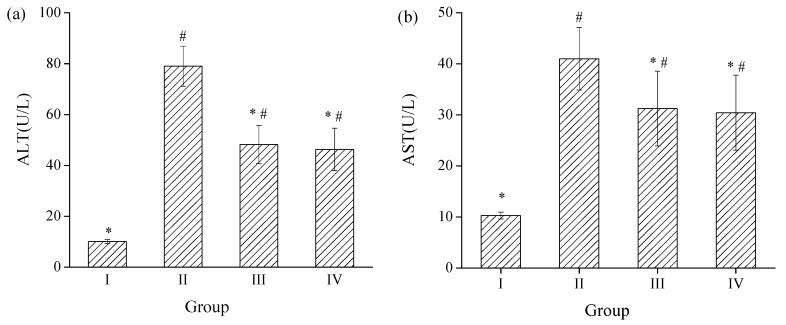
Effects of SPH-I on the activities of serum (**a**) alanine aminotransferase (ALT) and (**b**) aspartate aminotransferase (AST). Group I, normal control; Group II, alcohol model; Group III, SPH-I (100 mg/kg BW) + alcohol; Group IV, SPH-I (300 mg/kg BW) + alcohol; each group contained 10 KM mice.* Statistical significance *p* < 0.05, compared with alcohol-treated group. # Statistical significance *p* < 0.05, compared with control group.

The effects of SPH-I on the serum ALT and AST activities are shown in Figure 5. Mice with alcohol administration (groups II, III, and IV) showed a significant increase of serum ALT and AST activities compared with those of group I (*p* < 0.05) and the values of AST/ALT were less than 1, indicating that the alcohol-induced liver injury model in mice was well-established. Administration of SPH-I at 100 and 300 mg/kg BW revealed a significant protective effect on the alcohol-induced liver injury by attenuating the elevation of the activity of ALT by 38.9% and 41.4%, respectively (Figure 5a) and depressing the increase of the activity of AST by 23.8% and 25.8%, respectively, compared with the alcohol model group (Figure 5b).

#### 2.3.2. Effect of SPH-I on Hepatic MDA Level

**Figure 6:**
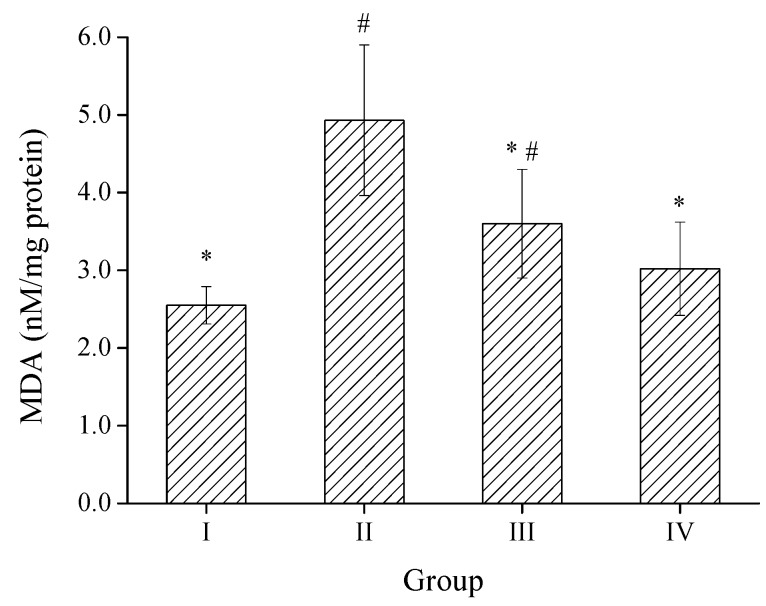
Effect of SPH-I on the hepatic malondialdehyde (MDA) level. Group I, normal control; Group II, alcohol model; Group III, SPH-I (100 mg/kg BW) + alcohol; Group IV, SPH-I (300 mg/kg BW) + alcohol; each group contained 10 Kunming (KM) mice. * Statistical significance *p* < 0.05, compared with alcohol-treated group. # Statistical significance *p* < 0.05, compared with control group.

MDA is the end-product of lipid peroxidation, whose levels could reflect the extent of cellular damage, serving as a marker of free radical-mediated lipid peroxidation [32]. Results shown in Figure 6 manifested that the hepatic MDA level of group II was remarkably enhanced after exposure to alcohol by 93.3%, indicating oxidative damage to the liver. Treatment of mice with SPH-I at the doses of 100 and 300 mg/kg significantly reversed the elevation of MDA levels by 27.0% and 38.7%, respectively, compared to the alcohol model (group II), suggesting that SPH-I could inhibit alcohol induced lipid peroxidation in the liver.

#### 2.3.3. Effects of SPH-I on Hepatic SOD, CAT, GSH-Px Activities, and GSH Level

**Figure 7:**
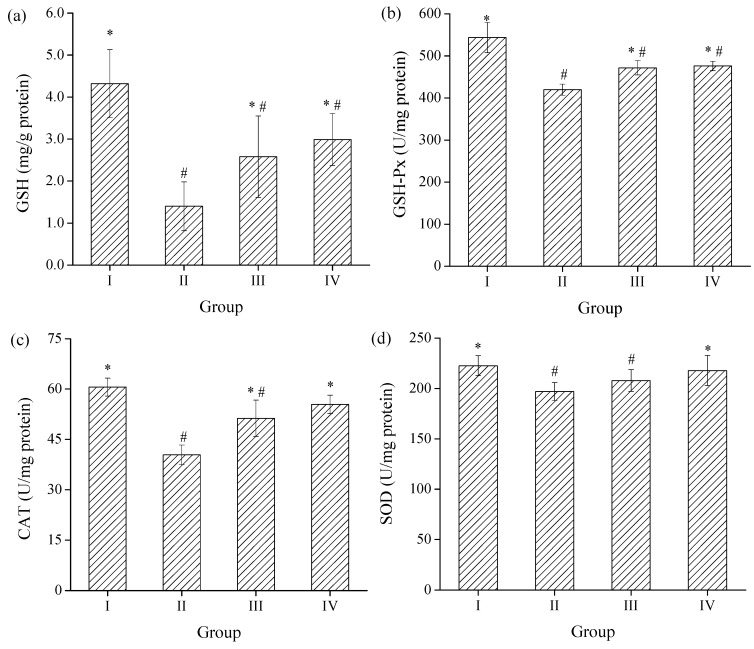
Effects of SPH-I on the level of hepatic (**a**) glutathione (GSH) level and (**b**) glutathione peroxidase (GSH-Px), (**c**) superoxide dismutase (SOD), (**d**) catalase (CAT) activities. Group I, normal control; Group II, alcohol model; Group III, SPH-I (100 mg/kg BW) + alcohol; Group IV, SPH-I (300 mg/kg BW) + alcohol; each group contained 10 KM mice.* Statistical significance *p* < 0.05, compared with alcohol-treated group. # Statistical significance *p* < 0.05, compared with control group.

Antioxidant enzymes play important roles in elimination of ROS derived from the redox reactions of xenobiotics in liver [33]. SOD is an efficient enzyme that catalyzes the conversion of superoxide into O_2_ and H_2_O_2_, and H_2_O_2_ could be further decomposed into H_2_O and O_2_ by CAT, GSH-Px, and the participation of GSH [34]. As a main non-enzymatic antioxidant in cells, GSH plays a critical role in antioxidant defense to protect cells from oxidative damage of ROS such as hydroxyl radical, lipid peroxyl radical, and H_2_O_2_ [35]. The effects of SPH-I on hepatic SOD, CAT, GSH-Px activities and GSH level were shown in Figure 7. Compared to the control group, the GSH level and GSH-Px, CAT, SOD activities were significantly decreased after exposure to alcohol by 67.6%, 22.8%, 33.3%, and 11.5%, respectively. The levels of GSH were 45.7% and 114% higher than those of group II with administration of SPH-I at the doses of 100 and 300 mg/kg BW respectively. Pretreatment of mice with SPH-I could also remarkably increase the hepatic SOD, CAT, and GSH-Px activities at the same time (*p* < 0.05), indicating that the hepatoprotective effects of SPH-I against acute alcohol-induced liver injury were due to the stabilization of intracellular antioxidant defense systems.

Previous report showed that a peptide from duck skin byproducts hydrolysate with strong free radical scavenging activities could inhibit the production of ROS and cell death against alcohol-induced liver cell damage, and enhanced the antioxidative enzymes (SOD, CAT, GSH-Px) activities in response to alcohol-induced oxidative damage in rats [36]. The antioxidant activity of an antioxidant compound has been attributed to various mechanisms, among which are radical scavenging, binding of transition metal ion catalysts, reductive capacity, prevention of chain initiation, decomposition of peroxides, and prevention of continued hydrogen abstraction [37]. The results obtained from the present study clearly validated powerful antioxidant activity of SPH-I against various oxidation systems in vitro, which contributed to its hepatoprotective effects of SPH-I in alcohol-induced liver injury in mice.

## 3. Materials and Methods

### 3.1. Materials

*Schizochytrium* processing byproduct was kindly provided by Fisheries Research Institute of Fujian, China, and was stored at −20 °C before use. The commercial protease, alcalase (EC. 3.4.21.62, 2.2 × 10^5^ U/g) and flavourzyme (EC. 3.4.11.1, 7.8 × 10^4^ U/g) were purchased from Novozymes (Copenhagen, Denmark). 2,2′-azinobis-3-ethylbenzthiazoline-6-sulphonate (ABTS), 1,1-diphenyl-2-picrylhydrazyl (DPPH) were obtained from Sigma Chemical Co. (St. Louis, MO, USA). All the kits for biochemical analyses used in the animal experiment were the products of Nanjing Jiancheng Bioengineering Institute (Nanjing, China). All other chemicals and reagents were of analytical grade and commercially available.

### 3.2. Preparation of SP

SP was extracted by using alkaline extraction and acid precipitation as described previously [38]. The *Schizochytrium* byproduct was ground to powder (sieved through a 50 mesh sieve). One percent (*w*/*v*) *Schizochytrium* powder in 0.39 M NaOH solution was stirred at 90 °C for 30 min and then centrifuged at 11,000× *g*, 20 °C for 20 min. The supernatant was adjusted to pH 3.0 by 6 M HCl solution and kept for 30 min (pH 3.0 was confirmed to precipitate most of the protein from the alkaline extract in our preliminary experiments). The mixture was centrifuged at 11,000× *g*, 20 °C for 20 min. The precipitated SP was lyophilized for further enzymatic hydrolysis.

### 3.3. Preparation of SPH

SPH was prepared through stepwise enzymatic hydrolysis by alcalase and flavourzyme sequentially. Two percent (*w*/*v*) SP was first hydrolyzed by alcalase at a ratio of alcalase to SP of 10% (*w*/*w*), pH 9.0 at 50 °C for 6 h. Then the mixture was hydrolyzed for another 8 h at 50 °C, pH 6.7 by flavourzyme (the ratio of flavourzyme to SP was 12.5%, *w*/*w*). The hydrolysate was heated at 100 °C for 10 min to inactive the enzymes and then cooled to room temperature. The SPH in the supernatant was collected by centrifugation at 11,000× *g* for 20 min, and then lyophilized and stored at −20 °C for further analysis.

### 3.4. Analysis of Amino Acid Composition

The lyophilized hydrolysate was digested at 110 °C for 24 h with HCl (6 M) under nitrogen atmosphere. A High Speed Amino Acid Analyzer Model L-8900 (Hitachi High-Technologies Co., Tokyo, Japan) was used to analyze the amino acid composition of the hydrolysate.

### 3.5. Determination of MW Distribution of SPH

MW distribution of SPH was determined using HPLC. The sample was applied to a Waters 650E Advanced Protein Purification System (Waters Corporation, Milford, MA, USA) equipped with TSKgel2000 SWXL column (300 mm × 7.8 mm). The mobile phase was 45% acetonitrile and 55% deionized water containing 0.1% trifluoroacetic acid. Chromatographic analysis was carried out with a flow rate of 0.5 mL/min and a column temperature at 30 °C. The absorbance was monitored at 220 nm. A calibration curve was obtained with bovine carbonic anhydrase (29,000 Da), horse heart cytochrome C (12,500 Da), aprotinin (6500 Da), bacitracin (1450 Da), gly–gly–tyr–arg (451 Da) and gly–gly–gly (189 Da). With the help of elution time of calibration materials, the linear regression equation was obtained for the calculation of MW. The results were processed with Millennium32 version 3.05 (Waters Corporation, Milford, MA, USA).

### 3.6. Ultrafiltration of SPH

SPH obtained from alcalase and flavourzyme digestion was fractionated through ultrafiltration membrane with a MW cut-off of 3 kDa (Millipore, Billerica, MA, USA). All fractions recovered were collected as SPH-I (MW < 3 kDa) and SPH-II (MW > 3 kDa).

### 3.7. Detemination of Antioxidant Activity In Vitro

#### 3.7.1. DPPH Radical Scavenging Activity

The scavenging activity of SPH and its fractions against DPPH radical was tested according to the method of Wu et al. [39] with slight modification. DPPH was dissolved in ethanol to a final concentration of 0.1 mM. 1 mL of sample was mixed with 1 mL of DPPH solution and then kept in the dark for 30 min at room temperature. Distilled water instead of the sample was used for control. The absorbance values of samples and control were measured at 517 nm. The scavenging rate of DPPH radical of the sample was evaluated with the following equation:

(1) $$\mathrm{DPPH}\;\mathrm{radical}\;\mathrm{scavenging}\;\mathrm{activity}\;(\%)\;=\;(A_{\mathrm{control}}-A_{\mathrm{sample}})/A_{\mathrm{control}}\times 100,$$

where *A*_sample_ and *A*_control_ were the absorbances of sample and control group, respectively.

#### 3.7.2. ABTS Radical Scavenging Activity

The ABTS radical scavenging assay was carried out according to the method of Wang et al. [24]. The ABTS radical was generated by mixing ABTS stock solution (7 mM) with equal volume of potassium persulfate (2.45 mM), and the mixture was incubated in the dark at room temperature for 12–16 h. The ABTS radical solution was diluted in phosphate buffer (5 mM, pH 7.4) to an absorbance of 0.70 ± 0.02 at 734 nm before used. 1 mL ABTS radical solution was added to 1 mL sample solution. The mixture was then incubated in the dark for 10 min and the absorbance was read at 734 nm. Distilled water instead of the sample was used for control. The ABTS radical scavenging activity of the samples was calculated by the following equation:

(2) $$\mathrm{ABTS}\;\mathrm{radical}\;\mathrm{scavenging}\;\mathrm{activity}\;(\%)\;=\;(A_{\mathrm{control}}-A_{\mathrm{sample}})/A_{\mathrm{control}}\times 100,$$

where *A*_sample_ and *A*_control_ were the absorbances of sample and control group, respectively.

#### 3.7.3. Reducing Power

The reducing power of SPH and its fractions was estimated according to Oyaizu [40] with some modification. 1 mL sample was mixed with 1 mL of phosphate buffer (0.2 M, pH 6.6) and 1 mL of 1% of potassium ferricyanide. The mixture was then incubated at 50 °C for 20 min followed by addition of 1 mL of 10% trichloroacetic acid. The mixture was then centrifuged at 2500× *g* for 10 min. 1 mL of the supernatant was mixed with 1 mL distilled water and 0.2 mL of 0.1% FeCl_3_. After 10 min, the absorbance was recorded at 700 nm.

#### 3.7.4. Inhibition of Linoleic Acid Peroxidation

The capacity of inhibiting linoleic acid peroxidation of SPH and its fractions was measured according to the method described by Osawa and Namiki [41] with some modification. Briefly, samples were dissolved in distilled water to a concentration of 1 mg/mL and then mixed with 2 mL of ethanol, 26 μL of linoleic acid and 2 mL of phosphate buffer (50 mM, pH 7.0). The mixture was incubated in a colorimetric tube with plug at 40 °C in the dark. The degree of oxidation was measured at 24 h intervals using the ferric thiocyanate (FTC) method of Mitsuda et al. [42]. 100 μL of the reaction mixture was added to a solution of 4.7 mL of 75% ethanol, 0.1 mL of 30% ammonium thiocyanate, and 0.1 mL of 20 mM FeCl_2_ solution in 3.5% of HCl. After 3 min, the degree of color development that represented the linoleic acid oxidation was measured spectrophotometrically at 500 nm.

### 3.8. Evaluation of Hepatoprotective Effects of SPH-I in Mice

#### 3.8.1. Animals and Treatments

Forty male KM mice with body weight (BW) of about 20 g were purchased from Slac Laboratory Animal Center (Shanghai, China). Throughout the experiments, mice were fed with standard pellet laboratory animal feed and had free access to food and water. The experiments were carried out in accordance with the guidelines issued by the Ethical Committee of Fujian Medical University (Fujian, China).

After a seven-day period of acclimatizing, forty mice were randomly divided into four groups with ten mice in each group. Group I served as normal control. Group II was alcohol model group in which mice were treated with alcohol alone. Group III and IV were mice treated with SPH-I at 100 and 300 mg/kg BW respectively for 24 consecutive days (the test dosages of SPH-I were decided by preliminary tests). Mice in group I and II were orally given the same volume of deionized water instead of SPH-I solution. One hour after substances administration at the 24th day, the mice except the normal control group were treated with single dose of 50% of alcohol (12 mL/kg BW), while group I was treated with the same volume of water. Whole blood was collected after a fasting period of 24 h. The mice were then euthanized and their livers were excised.

#### 3.8.2. Analysis of Serum Biochemical Indices

Blood samples were collected immediately and the serum was separated by centrifugation at 1500× *g* for 10 min at 4 °C. The activities of serum ALT and AST were analyzed using ALT and AST assay kits according to the manufacturer’s protocol.

#### 3.8.3. Analysis of Hepatic Biochemical Indices

Liver tissues were excised and homogenized in 0.1 g/mL of cold normal saline. The supernatant of the homogenate was collected after centrifugation at 3000× *g* for 10 min at 4 °C. The activities of SOD, CAT, GSH-Px, and the level of GSH and MDA were determined with T-SOD assay kit (hydroxylamine method), CAT assay kit (visible light), GSH-PX assay kit (colorimetric method), GSH assay kit, and MDA assay kit (TBA method) according to the manufacturer’s protocol, respectively. The total protein content of liver homogenate was determined according to the Bradford method [43].

### 3.9. Statistical Analysis

All results are presented as means ± standard deviation (SD). Statistical analysis was carried out with IBM SPSS 17.0 software (SPSS, Chicago, IL, USA). Statistical analysis was performed by one-way analysis of variance (ANOVA) with Duncan’s test for post hoc analysis and *p* < 0.05 values were considered as statistically significant.

## 4. Conclusions

In this study, *Schizochytrium* sp. protein hydrolysate was prepared by alcalase and flavourzyme sequentially, and mainly composed of Glu (17.66%), Asp (15.89%), Leu (9.96%), and Arg (7.81%) along with small amounts of Phe (5.27%), Tyr (2.71%), and His (1.57%). After ultrafiltration of SPH with 3 kDa membrane, SPH-1, peptides with MW below 3 kDa, exhibited the highest DPPH and ABTS radicals scavenging activities, reducing power and lipid peroxidation inhibition potential. In addition, SPH-I could significantly alleviate alcohol-induced hepatotoxicity in mice. Results of the present study indicated that SPH-I could be developed as a potential functional antioxidant additive for effective therapy of alcohol-induced liver diseases.

## Figures and Tables

**Table 1:** Amino acid composition of *Schizochytrium* protein hydrolysate (SPH).

Amino Acids	Content (%)
Ile	3.52
Leu	9.96
Met	1.35
Phe	5.27
Thr	3.93
Val	5.17
Lys	5.79
Cys	0.62
Tyr	2.72
Asx ^a^	15.89
Ser	5.42
Glx ^b^	17.66
Gly	3.88
Ala	5.28
Pro	4.16
Arg	7.81
His	1.57
THAA ^c^	34.71
Total	100

^a^ Asx: containing Asp and Asn; ^b^ Glx: containing Glu and Gln; ^c^ THAA: total hydrophobic amino acid.
